# Patient Outcomes Following Reduction and Association of the Scaphoid and Lunate: A Retrospective Cohort Study

**DOI:** 10.1055/a-2537-2648

**Published:** 2025-03-06

**Authors:** Zoe E. Mack, Brodie Ritchie, Adina Tarcea, Gurpreet S. Dhaliwal, Neil J. White

**Affiliations:** 1 South Campus Research Unit for Bone and Soft Tissue, Department of Orthopedic Surgery, University of Calgary, Calgary, Alberta, Canada

**Keywords:** RASL, scapholunate ligament disruption, scapholunate, carpal instability

## Abstract

**Background:**

Scapholunate interosseous ligament (SLIL) injury is a common ligamentous injury of the wrist; however, the optimal operative management strategy remains unclear. The objective of this study was to investigate patient outcomes following the Reduction and Association of the Scaphoid and Lunate (RASL) procedure.

**Materials and Methods:**

Twenty-five consecutive patients who had an SLIL tear treated with RASL completed a demographic survey and three standardized patient-reported outcome tools (Disabilities of the Shoulder, Arm and Hand [DASH], Patient-Rated Wrist Evaluation [PRWE], and Patient Reported Outcome Measurement Information System, Upper Extremity [PROMIS] questionnaires). Standard wrist radiographs were taken preoperatively and postoperatively and bilateral wrist range of motion was measured.

**Results:**

At an average postsurgical time of 4.6 years, the average DASH score was 10.5 with a right-skewed distribution. There was no correlation between screw angle, preoperative scapholunate angle, or time from surgery and DASH score.

**Conclusion:**

We conclude that with meticulous surgical technique, patient reported and radiographic outcomes demonstrate the relative success of the RASL procedure as a viable option for SLIL reconstruction in appropriate candidates.

**Level of Evidence:**

Level IV evidence—a retrospective cohort study.

The problem of managing scapholunate ligament (SLIL) injury remains unsolved, with multiple new techniques emerging each year. It has been well-established that without treatment, patients can develop a progressive pattern of dorsal intercalated segment instability (DISI), followed by a progression to scapholunate advanced collapse (SLAC). 1 Numerous surgical approaches have been proposed to treat this condition; however, the optimal solution remains unclear. 2 3

One promising technique for SLIL reconstruction is the Reduction and Association of the Scaphoid and Lunate (RASL) procedure, first described by Rosenwasser et al. 4 In this procedure, a screw is inserted distally through the scaphoid and crosses obliquely into the proximal ulnar corner of the lunate to reassociate the scaphoid and lunate. Despite promising outcomes by the creator, 5 other studies have demonstrated variability. 6 7 8 Given the limited number of studies and small sample sizes, it is difficult to understand this variability in outcomes. Contributing factors may include patient selection, injury characteristics, or technical challenges associated with screw placement. Incorrect placement distal to the lateral aspect of the dorsal scaphoid ridge has been associated with failure in a biomechanical cadaveric study. 9

Understanding the factors affecting patient-reported outcomes following the RASL procedure can help explain the variability in patient outcomes and identify good surgical candidates. The objective of this study was to retrospectively assess the outcomes of the RASL procedure in a series of consecutive patients from 2012 to 2023. We hypothesized that preoperative factors including increased scapholunate angle and scapholunate gap would be associated with poor patient-reported outcomes and postoperative complications.

## Materials and Methods

### Subject Selection and Data Collection

We performed a retrospective review of all patients who underwent a primary RASL procedure performed by two surgeons from a single center (N.J.W. and G.S.D.) between January 2012 and January 2023. After institutional board review and Research ethics board's (REB) approval, Health Service Codes were used to identify potential participants. Using this method, patients who had undergone a “Wrist Ligament Reconstruction” (Fee Code 93.87k) were identified and reviewed for inclusion in our study.

**Fig. 1:**
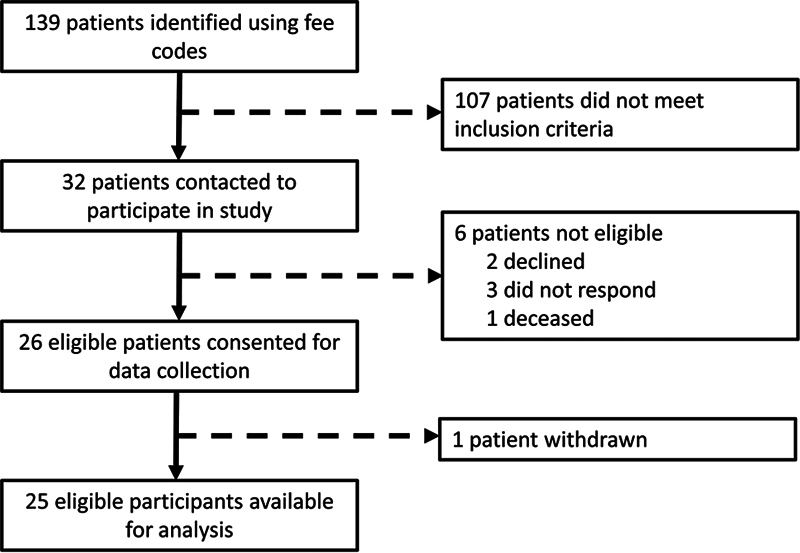
Diagram summarizing the process of participant selection. In total, 139 patients who had a wrist ligament reconstruction were identified in two rounds of screening. A total of 107 participants were screened out based on the nature of surgery (i.e., had ligament reconstruction other than a primary RASL), surgical technique, or failure to meet other inclusion criteria. RASL, Reduction and Association of the Scaphoid and Lunate.

All patients with a complete SLIL tear who underwent repair using the RASL procedure from January 2013 and January 2024 and agreed to participate were recruited to the study ( Fig. 1 ). The recruitment and data collection period spanned from January 2022 to January 2024. Patients were required to be a minimum of 1 year postsurgery at the time of data collection. We excluded patients who had a concomitant fracture or dislocation of another ipsilateral carpal bone, distal radius, or ulna, and patients with a history of musculoskeletal disorders such as fibrous dysplasia, chronic renal failure, Paget's disease, or osteopetrosis. Patients who were unable to provide adequate questionnaire answers or undergo radiographic imaging due to medical or cognitive comorbidities were also excluded.

Patients who satisfied all the inclusion criteria and exclusion criteria were contacted to participate in our study. Those who consented to participate were asked to fill out a demographic survey ( **Appendix 1** [available in the online version only]), which included questions regarding the cause of injury and functional status before and after the surgery. Patients were also asked to complete a series of questionnaires, including the Disabilities of the Shoulder, Arm and Hand (DASH) questionnaire, the Patient-Rated Wrist Evaluation (PRWE) questionnaire, and the Patient Reported Outcome Measurement Information System, Upper Extremity (PROMIS). Standard wrist radiographs were obtained for all participants. Participants were asked to return for an inpatient visit during which bilateral wrist range of motion (ROM) was measured using a goniometer and bilateral hand strength was measured. Two participants were unable to attend this visit and therefore were excluded from the ROM analysis. The participant selection process is summarized in Fig. 1 . One participant withdrew from the study after consent was obtained and therefore their data were excluded from the analysis. Study data were collected and managed using REDCap (Research Electronic Data Capture) electronic data capture tools. 10 11 REDCap is a secure, web-based software platform designed to support data capture for research studies, providing (1) an intuitive interface for validated data capture; (2) audit trails for tracking data manipulation and export procedures; (3) automated export procedures for seamless data downloads to common statistical packages; and (4) procedures for data integration and interoperability with external sources. 10

### Surgical Technique

Candidates for the RASL procedure included patients with chronic or subacute SLIL ligament tears without evidence of arthritis on MRI or wrist arthroscopy, as per the standard practice of the treating surgeons. Where possible, the procedure was performed in a staged manner, with diagnostic arthroscopy to confirm eligibility for the procedure followed by open RASL. All patients underwent an open RASL procedure by one of two fellowship-trained hand surgeons using a dual-incision approach as described by Rosenwasser et al. 4 Care was taken to ensure adequate debridement was performed between the scaphoid and lunate with a combination of a high-speed burr to penetrate the cartilage and angled curettes for a controlled C-shape debridement following the contour of the bones. The debridement was taken down to expose cancellous bone on both the scaphoid surface of the lunate and the lunate surface of the scaphoid. The use of angled cervical curettes allows for a controlled debridement without the risk of injuring the cartilage surface of the capitate. Joystick k-wires allow derotation of the scaphoid and lunate. Reduction is confirmed visually and with fluoroscopy. In all cases, the first extensor compartment was released and a radial styloidectomy was performed. Care was taken to obtain a screw trajectory through the center of the scaphoid and lunate aiming obliquely from the scaphoid to the proximal ulnar corner of the lunate. All cases used a stainless steel 3.0-mm smooth shafted cannulated headless compression screw (Synthes). Operative reports were reviewed for all patients to confirm the homogeneity of the surgical technique.

### Radiographic Assessment and Data Analysis

A chart review was performed to identify preoperative and intraoperative or immediately postoperative (within 2 weeks) radiographs of each participant's affected wrist for comparison with the long-term follow-up radiographs. The scapholunate interval, scapholunate angle, radioscaphoid angle, and radiolunate angle were measured on standardized anteroposterior (AP) and lateral radiographs preoperatively and at the time of long-term follow-up (minimum 1-year postsurgery). The intraoperative images were used to assess screw placement. All measurements were taken manually by the primary author and independently verified by two board-certified hand surgeons. Data analysis was performed in MATLAB R2021b (Mathworks, Natick, MA). A one-tailed *t* -test was used to compare the scapholunate angle and scapholunate gap between the preoperative, postoperative, and follow-up radiographs. A linear correlation coefficient was used to assess for relationship between radiographic measurements and patient-reported outcome measures.

## Results

### Participant Demographics

Of the 32 consecutive patients contacted, 25 eligible participants (17 male) completed data collection ( Fig. 1 ). The average time postsurgery was 4.6 (SD 2.8) years at the time of follow-up, with a minimum time since surgery of 1.0 years and a maximum of 9.6 years. The average time between injury and surgery was 1.5 (SD 2.2) years. The average age at the time of injury was 36.9 (SD 10.7) years ( Table 1 ). The most common mechanism of injury was sport-related injury ( *n* = 16), followed by ground-level fall ( *n* = 5), blunt trauma ( *n* = 3), and other mechanism ( *n* = 1). The diagnosis of SLIL tear was confirmed by either arthroscopy ( *n* = 14), MRI ( *n* = 7), or using plain radiographs ( *n* = 4) with diagnostic arthroscopy at the time of surgical intervention. On preoperative radiographs, 16 of 25 participants had a DISI deformity (defined as a scapholunate angle greater than 60 degrees), and 8 participants had dorsal subluxation of the scaphoid.

**Table 1:** Summary of participant demographics and injury characteristics

Demographic variable	Value
**Gender**	17 male, 8 female
**Age (years)**	37.0 years (SD 10.7)
**BMI**	27.4 (SD 4.9)
**Smoking** s **tatus**	3 smokers, 22 non-smokers
**Dominant** h **and**	24 right, 1 left
**Dominant** hand injured	16 participants

Abbreviations: BMI, body mass index; SD, standard deviation.

### Range of Motion and Grip Strength

**Fig. 2:**
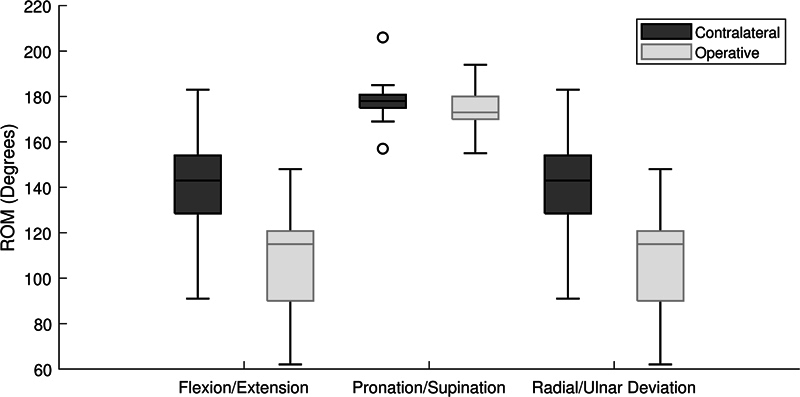
Postoperative range of motion of the operative hand compared with the contralateral (control) hand. There was a significant difference ( *p* < 0.05) between the range of motion of the injured and control side in flexion–extension, pronation–supination, and radial–ulnar deviation.

Among the 24 of 26 participants who completed the ROM measurements, participants' average ROM of the operative side at the time of long-term follow-up was 108.7 degrees (SD 22 degrees) in flexion–extension, 173.3 degrees (SD 9 degrees) in pronation–supination, and 56.6 degrees (SD 14 degrees) in radial–ulnar deviation. Figure 2 displays the ROM of the operative side as compared with the ROM of the contralateral side. At the time of follow-up, patients' motion was decreased on the affected side in flexion–extension (78% contralateral, *p* < 0.001), radial–ulnar deviation (77% contralateral, *p* < 0.001), and pronation and supination (97% contralateral, *p* = 0.008; Fig. 2 ). The mean grip strength of the operative side was 95% of the contralateral side ( *p* < 0.001). Of the 24 participants who were able to perform pushups or a “downward dog” yoga pose before their injury, 17 participants regained the ability to perform these activities using modifications if needed after surgery, while 7 participants were no longer able to do pushups or downward dog pose at all.

### Radiographic Outcomes

A one-tailed *t* -test confirmed a significant difference between the measured scapholunate angle and the gap at the preoperative and follow-up visits, as well as a significant difference in the scapholunate angle between the intraoperative and follow-up measurements ( *p* < 0.05) ( Table 2 ), showing that the correction in the scapholunate angle is partially lost over time. The scapholunate gap was not able to be accurately assessed on intraoperative fluoroscopy images as the images were not calibrated. However, there was no correlation between the final scapholunate angle, final scapholunate gap, or screw angle and patient-reported outcome scores (DASH, PRWE, or PROMIS; *R* ^2^ < 0.1). The average angle of the screw placed between the scaphoid and lunate was 15.4 degrees (SD 6.3 degrees).

**Table 2:** Average radiographic measurements

	Preoperative	Intraoperative	Follow up
**SL angle (deg)**	65.8 (SD 10.3) a	47.5 (SD 9.1) a	60.0 (SD 12.9) a
**SL gap (mm)**	3.2 (SD 1.4) a		2.4 (SD 1.1) a
**RL angle (deg)**	−11.1 (SD 13.9)		−10.2 (SD 12.7)
**RS angle (deg)**	53.4 (SD 15.6)		52.4 (SD 18.7)

Abbreviations: deg, degrees; SD, standard deviation; SL, scapholunate.

a One-tailed *t* -test demonstrated a significant difference in SL angle between preoperative and follow-up radiographs ( *p* = 0.03), between intraoperative and follow-up radiographs ( *p* < 0.005), and between intraoperative and follow-up SL angle measurements ( *p* < 0.005). There was also a significant difference in SL gap between preoperative and follow-up radiographs ( *p* = 0.005).

### Patient-reported Outcomes

**Fig. 3:**
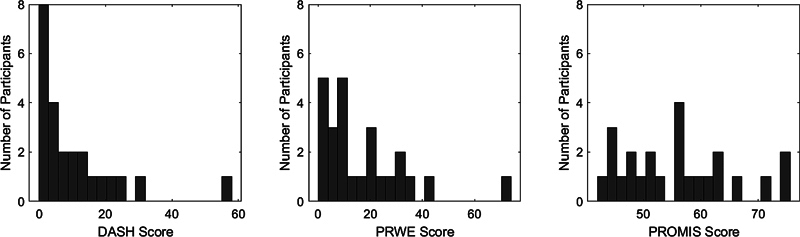
Distribution of DASH, PRWE, and PROMIS scores. DASH, Disabilities of the Shoulder, Arm and Hand; PROMIS, Patient Reported Outcome Measurement Information System, Upper Extremity; PRWE, Patient-Rated Wrist Evaluation.

The average DASH score at the time of follow-up was 10.5 (SD 13), with a range of 0 to 57.5 in a right-skewed distribution ( Fig. 3 ). There was no correlation between DASH score and time from surgery to final follow-up ( *R* ^2^ = 0.04). A two-sample *t* -test confirmed there was no significant variability in DASH score between surgeons ( *p* = 0.11). There was a positive linear correlation between DASH and PRWE scores ( *R* ^2^ = 0.75, *p* < 0.005). There was no correlation between any patient-reported outcome measure (including DASH, PROMIS, or PRWE score) and preoperative scapholunate angle or preoperative scapholunate gap ( *R* ^2^ < 0.1).

### Complications

Two intraoperative complications were reported, including overcorrection of DISI deformity following broken k-wires, and a fracture of the proximal pole of the scaphoid ( Table 3 ). All participants were included in the analysis according to intention to treat. One participant subsequently underwent a salvage procedure (four-corner fusion) following the failure of the RASL.

**Table 3:** Summary of intraoperative complications

Age	Sex	Description of complication	Preoperative SL gap	Preoperative SL angle	DASH score
**30**	M	Broken k-wires, overcorrection of DISI deformity	3.6	51.0	22.5
**47**	F	Fracture of proximal pole of scaphoid	4.4	72.9	6.6

Abbreviations: DASH, Disabilities of the Shoulder, Arm and Hand; DISI, dorsal intercalated segment instability; SL, scapholunate.

### Screw Removal

Four participants underwent a second surgery for hardware removal ( Table 4 ). The average time between surgery and hardware removal was 1.7 years (SD 2.2). One screw was removed electively for styloscaphoid pain, and three were removed at the surgeon's recommendation because of excessive lucency around the screw head in the scaphoid. There was no significant difference in DASH scores at the time of final follow-up between patients who underwent hardware removal and those who did not ( *p* = 0.14).

**Table 4:** Summary of demographics and outcomes in the revision group

Age	Sex	BMI	Smoking status	Description of revision	Preoperative SL gap	Preoperative SL angle	DASH score
**41**	M	31.4	Non-smoker	4-corner fusion	5.6	76.1	57.5
**30**	M	30.1	Non-smoker	Hardware removal—patient-driven	6.8	74.9	5.0
**42**	F	39.9	Non-smoker	Hardware removal—surgeon-driven	1.9	48.3	0.8
**47**	F	19.6	Non-smoker	Hardware removal—surgeon-driven	4.4	72.9	6.7
**19**	M	26.6	Smoker	Hardware removal—surgeon-driven	6.5	77.9	0

Abbreviations: BMI, body mass index; DASH, Disabilities of the Shoulder, Arm and Hand; SL, scapholunate.

## Discussion

Twenty-five patients with an SLL tear treated with RASL procedure who met our inclusion criteria completed data collection, with an average age of 36.9 (SD 10.7) years at the time of injury. All patients underwent an open RASL procedure using a standardized technique involving a dual-incision approach with debridement between the scaphoid and lunate to expose cancellous bone on both the scaphoid surface of the lunate and lunate surface of the scaphoid prior to screw placement. The average time since surgery was 4.6 years, with a minimum time of 1.0 years and a maximum time of 9.6 years. At the time of final follow-up, most patients reported good outcomes, with an average DASH score of 10.5. Most patients (17 out of 24) who were able to perform push-ups or downward dog pose prior to their injury regained the ability to perform these activities at the time of final follow-up, using modifications if needed.

The average ROM and DASH scores postoperatively were comparable to that seen in a similar investigation by Koehler et al 7 who reported an average DASH score of 8 with 46 degrees of wrist flexion and 56 degrees of extension (102 degrees flexion–extension arc) after arthroscopic RASL with a mean follow-up time of 36 months. The ROM was also comparable to an analysis by Caloia et al 12 , who found an average flexion–extension arc of 107 degrees postarthroscopic RASL, a 20% reduction compared with the contralateral side. 12

We found a significant difference between both the SL gap and SL angle preoperatively compared with postoperatively. The finding of improved SL gap is consistent with the findings of a cadaveric biomechanical study which showed that among three-ligament tenodesis, anatomic front and back reconstruction, and RASL; RASL was the only SLIL reconstruction technique that statistically improved scapholunate gap. 13 However, we also observed a statistically significant change in SL angle when compared with our intraoperative and follow-up measurements, suggesting the SL angle correction is partially lost over time. This is an expected outcome by the treating surgeons, who overcorrect the scapholunate angle at the time of surgery to account for loss of correction over time. This overcorrection may explain the maintained improvement in the SL angle seen in our results, which was not seen in a cohort of 12 patients followed by Aibinder et al, where the SL correction was completely lost over time. 6 Despite the radiographic loss of correction, the patient-reported outcomes revealed good function at the time of follow-up. A recent meta-analysis by Wu and Ilyas comparing SLIL reconstruction techniques reported an average DASH score of 24.4 for capsulodesis, 19.4 for tenodesis-based SLIL repair techniques, and 9.7 for a bone tissue bone approach. 14 RASL was not included in this analysis due to limited data. Our findings of an average DASH score of 10.5 indicate patient-reported outcomes comparable to the bone tissue bone approach (DASH 9.7).

The average screw angle measured on intraoperative radiographs was 15.4 degrees (SD 6.3 degrees), which falls below the target angle of 23 degrees as described by Rosenwasser et al 4 ; however, we did not find a correlation between screw angle and patient-reported outcomes as measured by the DASH, PRWE, or PROMIS. This suggests that there are likely factors other than screw trajectory in the AP plane influencing patient outcomes. A biomechanical study by Koehler et al found that screw placement distal to the lateral aspect of the dorsal scaphoid ridge was significantly associated with failure. 9 Taken in combination with our finding that the screw angle measured in the AP plane is not correlated with patient-reported outcome measures or revision rates, it is likely that there are additional factors in ideal screw placement, such as scaphoid morphology, that are not captured by measurement in a 2D plane.

Four patients underwent hardware removal at an average time of 1.8 years postop. Despite a secondary procedure, their DASH scores indicated an ultimately satisfactory outcome ( Table 4 ). One participant's postoperative course was complicated by a fall with resulting screw displacement which led to the failure of the RASL. A salvage procedure was performed (four-corner fusion), with a resulting DASH score of 57.5, the highest among our participant group ( Table 3 ). Koehler et al (2016) 7 reported that a preoperative SL gap of greater than 5 mm and the presence of SLAC Grade I were predictive of complication or revision surgery after RASL. The correlation between preoperative SL gap >5 mm and revision surgery was not seen among our cohort.

This investigation is limited by the small sample size and the retrospective study design. We do not have preoperative data to use as a point of comparison for ROM measurements or postoperative DASH, PROMIS, or PRWE scores. After 12 years of data collection, our analysis is limited to 25 patients, with all procedures performed by two surgeons from a single center. Despite the limited number of participants, it is worth noting that this patient cohort is similar in size or larger than as seen in previous works investigating RASL outcomes. 5 6 7 8 12 We hypothesize that the positive results shown here reflect the treating surgeons' practice pattern of carefully selecting appropriate candidates for the procedure. Given few patients meet these criteria, larger-scale studies become very challenging.

In summary, many patients with subacute or chronic SLIL injury without arthritis have excellent patient-reported outcomes following the RASL procedure; however, the population of ideal candidates for the procedure remains small. Possible factors contributing to patient outcomes include screw position, severity of the injury, and patient factors such as BMI or smoking status. With meticulous surgical techniques including adequate debridement between the scaphoid and lunate, accurate screw trajectory, and intraoperative overcorrection of the SL angle, patient-reported and radiographic outcomes demonstrate the relative success of the RASL procedure as a viable option for SLIL reconstruction in appropriate candidates.

## Figures and Tables

**Table:** 

Baseline demographics				
Hand dominance	❑ Right	❑ Left	
Gender	❑ Female ❑ Male ❑ Other _____________________			
Height	______________	❑ feet/inches	❑ centimeters
Weight	______________	❑ pounds	❑ kilograms
Age at follow-up	______________			
Occupation status			
Status of current employment ❑ Employed, please indicate job _____________________________________________ ❑ Unemployed ❑ Disabled ❑ Student ❑ Retired			
Is the current occupation active or sedentary? ❑ Active ❑ Sedentary ❑ N/A			
Status of employment at the time of injury ❑ Employed, please indicate job _______________________________________________ ❑ Unemployed ❑ Disabled ❑ Student ❑ Retired			
Was the occupation active or sedentary? ❑ Active ❑ Sedentary ❑ N/A Did you return to the same job prior to injury? ❑ Yes, full duties ❑ Yes, modified duties ❑ No ❑ N/A			
Can you complete pushups (and/or downward dog) with open palms? ❑ Yes, no modifications ❑ Yes, with modifications ❑ No ❑ I do not perform these activities.			
Were you able to complete pushups (and/or downward dog) with open palms prior to your injury? ❑ Yes, no modifications ❑ Yes, with modifications ❑ No ❑ Unknown			
Smoking status at the time of injury: ☐ Smoker ☐ Non-smoker			
Smoking status at the time of follow-up: ☐ Smoker ☐ Non-smoker			

## Notes

Appendix 1 Demographic questionnaire
